# Predoctoral MD-PhD grants as indicators of future NIH funding success

**DOI:** 10.1172/jci.insight.155688

**Published:** 2022-03-22

**Authors:** Shohini Ghosh-Choudhary, Neil Carleton, S. Mehdi Nouraie, Corrine R. Kliment, Richard A. Steinman

**Affiliations:** 1 Medical Scientist Training Program,; 2 Division of Pulmonary, Allergy, and Critical Medicine, Department of Medicine,; 3 Department of Medicine, and; 4 Department of Pharmacology and Chemical Biology, University of Pittsburgh School of Medicine, Pittsburgh, Pennsylvania, USA.

**Keywords:** Aging, Behavior

## Abstract

MD-PhD trainees constitute an important source of physician-scientists. Persistence on this challenging path is facilitated by success in garnering independent (R grant) support from the NIH. Published research tracks academic appointments and global R01 success for MD-PhD trainees but has not included information on future funding success of individual MD-PhD predoctoral grant holders. Here, we used data from the NIH RePORTER database to identify and track the funding trajectory of physician-scientists who received predoctoral grant support through the F30 mechanism, which is specific for dual-degree candidates. Male and female F30 awardees did not differ in their success in garnering K (postdoctoral training) grants, but, among F30 grant awardees, men were 2.6 times more likely than women to receive R funding. These results underscore the need for analysis of factors that contribute to the disproportionate loss of NIH-supported female physician-scientists between the predoctoral F30 and the independent R grant–supported stages.

## Graphical abstract

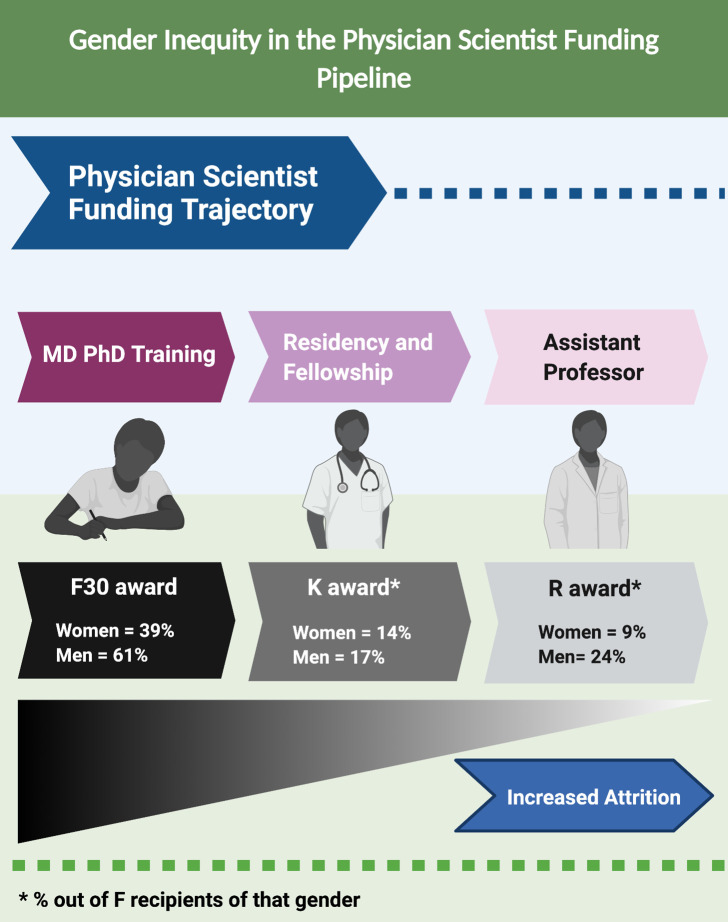

## Introduction

Physician-scientists have been a cornerstone of academic medicine and science and have pioneered basic, translational, and clinical discoveries to benefit patients’ lives. The concept of physician-scientists has been championed since the 1950s, beginning with the tenure of James Shannon, MD, as NIH Director. Many physicians engaged in research at NIH as an option to meet obligations of the physician draft from the Korean to the Vietnam War (2, 3). Since that time, there have been investments made to create a cadre of researchers whose work will be informed by clinical experiences with patients. The first National Institute of General Medical Sciences (NIGMS) Medical Scientist Training Program (MSTP) institutional T32 award for programs that combine graduate school with medical school was established in 1964 (4). This development sparked an era in which academic medical institutions and the NIH collaborated to invest in physician-scientist development through both medical school training and early-career physician-scientist development (1).

However, as soon as the late 1970s, leaders in medicine voiced concerns about physician-scientists as an “endangered species” (5). With these calls to action, the NIH, private foundations, and professional societies responded by increasing investments into physician-scientist training, including clinical research funding (K23, K24), loan repayment (LRP), and institutional (K30) awards, which were initiated between 1998 and 2002, and young investigator awards specifically geared to physician-scientists (6). Along these lines, a specific funding mechanism to support dual-degree candidates, the F30 National Research Service Award (NRSA), was first offered in 1990. This award was created to support dual-degree, mostly MD-PhD, candidates through their research training in medical school. Now, decades later, concern remains about the so-called “leakiness” of the physician-scientist training pipeline. Gender equity is an additional focus, as research has shown that gender differences in academic success and attainment of research funding opportunities continue to persist despite equal admission of women and men into medical school (7–12).

Knowledge gaps remain about the dynamics of attrition along the physician-scientist training pipeline (13–15). Moreover, the time points in this training path with the highest attrition rates need to be identified in order to develop a greater understanding of where targeted approaches for retention are needed. There have been important analyses of gender-based differences in the K-to-R award funding path (8, 9). To focus more on MD-PhD dual-degree training, we used publicly available data from the NIH RePORTER database (https://reporter.nih.gov/) to follow MD-PhD trainees, starting with the F30 NRSA fellowship award. It is conceivable that predoctoral F30 grant MD-PhD awardees, having demonstrated early competency to garner funding, will be better prepared to compete for future funding and to persist in academic careers independent of gender. We specifically focused on this group of dual-degree physician-scientists and subsequently analyzed the proportions and time to award for those who go on to receive K and R support, with analysis of gender differences at each of these time points.

## Results

### Overall F30 awards.

**Figure 1:**
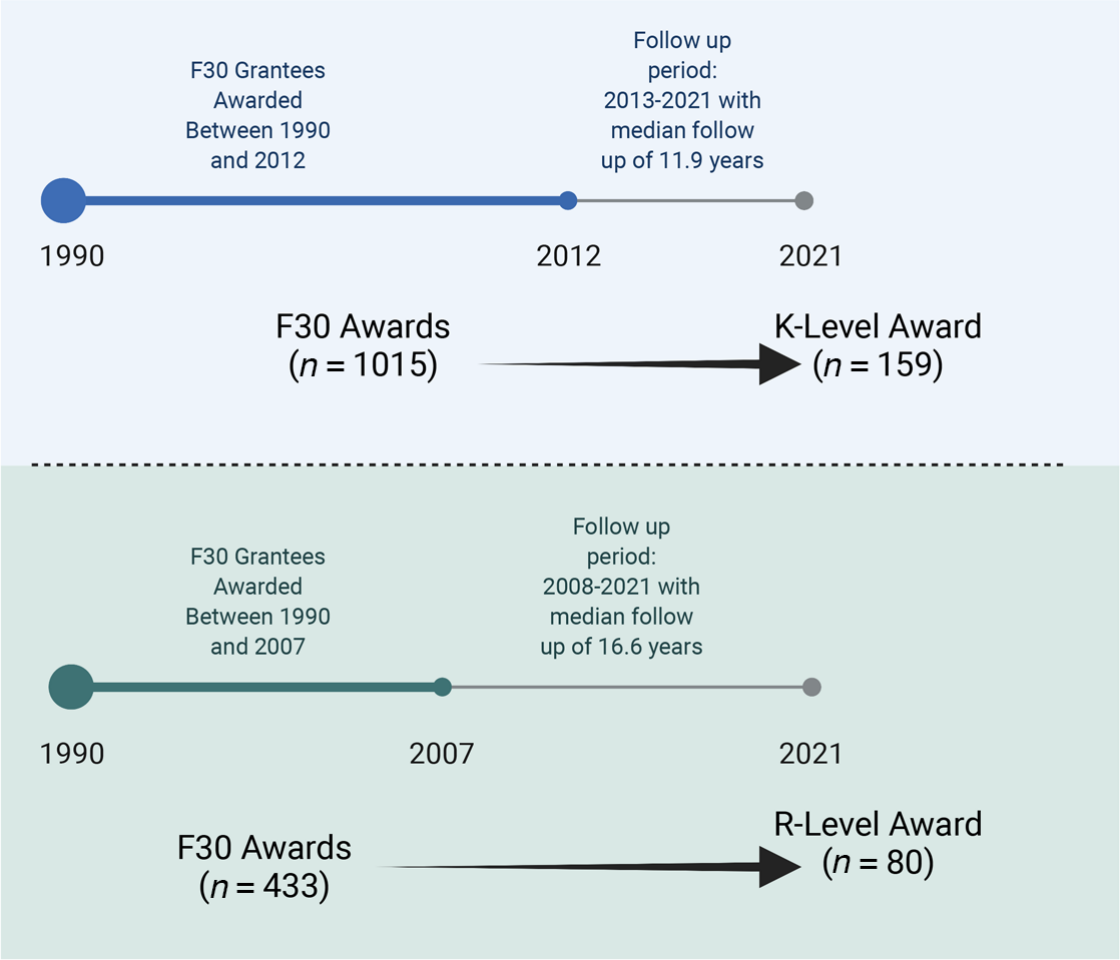
Schematic showing how analysis was performed. F30-to-K analysis (top) and the F30-to-R analysis (bottom).

We captured data on awardees receiving F30 awards between 1990 and 2012 (Figure 1). Over the course of this period, the number of institutions participating in this award increased, although most awards were garnered by students at a small number of institutions. From 1990 to 2007, 50% of F30 awards were accounted for by 14 institutions; from 2008 to 2012, 18 institutions accounted for 50% of the F30 awards.

**Figure 2:**
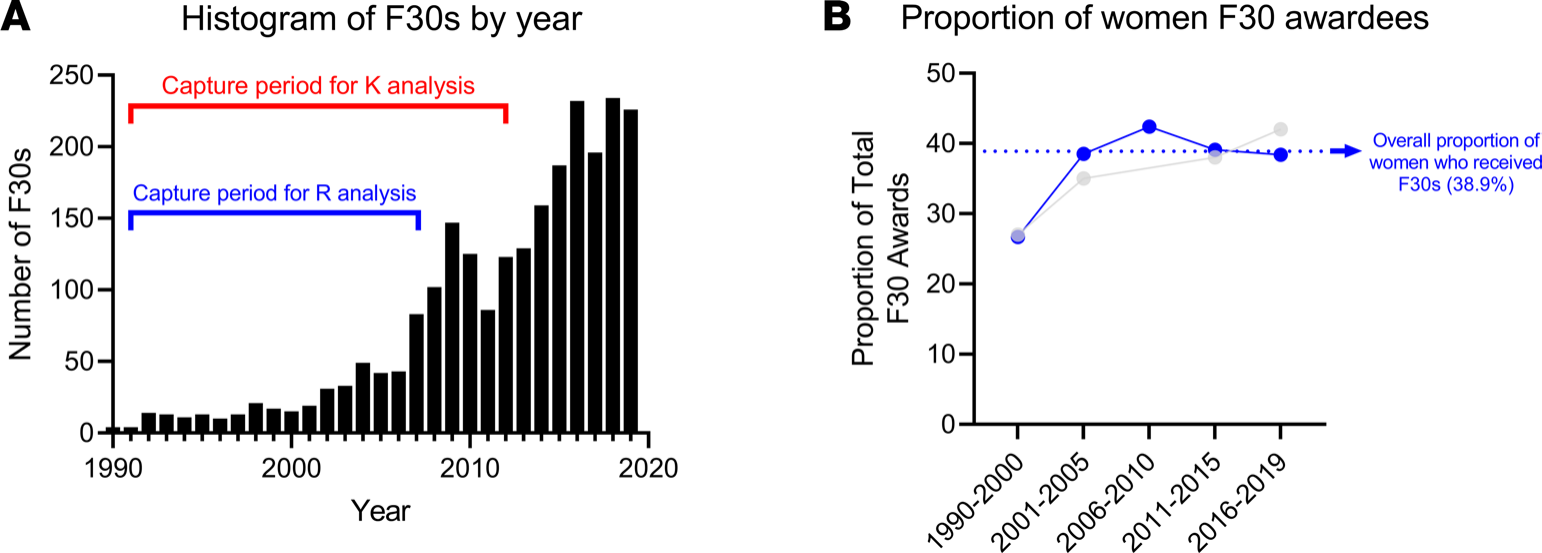
Distribution of F30 awards. (**A**) Overall histogram showing distribution of F30 awards to dual-degree MD-PhD trainees over time. (**B**) The blue line indicates the proportion of F30 recipients who were women by year. The overall proportion of women who received F30 awards was 38.9%; this percentage has increased from below 30% when the F30 awards were first awarded in the 1990s. We note that the proportion of female F30 awardees has remained level beyond the capture period for this study. These rates largely mirror the percentage of women who make up total enrollees in nationwide MSTP programs (indicated by light gray line) (note that the rates indicated for 1990–2000 and 2001–2005 are derived from Akabas and Brass, which show percentage of women graduates, which may differ slightly from the percentage of women enrollees, ref. 7; those for 2011–2019 are from AAMC, ref. 39).

We observed that the majority of F30 awards were granted in the past 10 years, many of which were beyond our K or R capture period due to the fact that we wanted to allow for adequate follow-up time (Figure 2A). Overall, when all years of F30 awards were combined, women constituted 38.9% of all awardees. This was comparable to the proportion enrolled in MD-PhD programs (Figure 2B).

### F30-to-K analysis.

F30 grantees who received their award between 1990 and 2012 were included in the F30-to-K analysis. Over this time period, there were 1015 F30 awardees, 404 (39.8%) of whom were women and 611 (60.2%) of whom were men. With a subsequent median follow-up period of 11.9 years for these F30 awardees, we found that 159 (15.7%) of the F30 awardees went on to receive a K-equivalent award, including 57 women (35.8% of K recipients) and 102 men (64.2%). With normalization, 14.1% of women who received an F30 award went on to receive a K award (57 of 404 total eligible); 16.7% of men who received an F30 award went on to receive a K award (102 of 611 eligible) (*P* = 0.29, Fisher’s exact testing).

**Figure 3:**
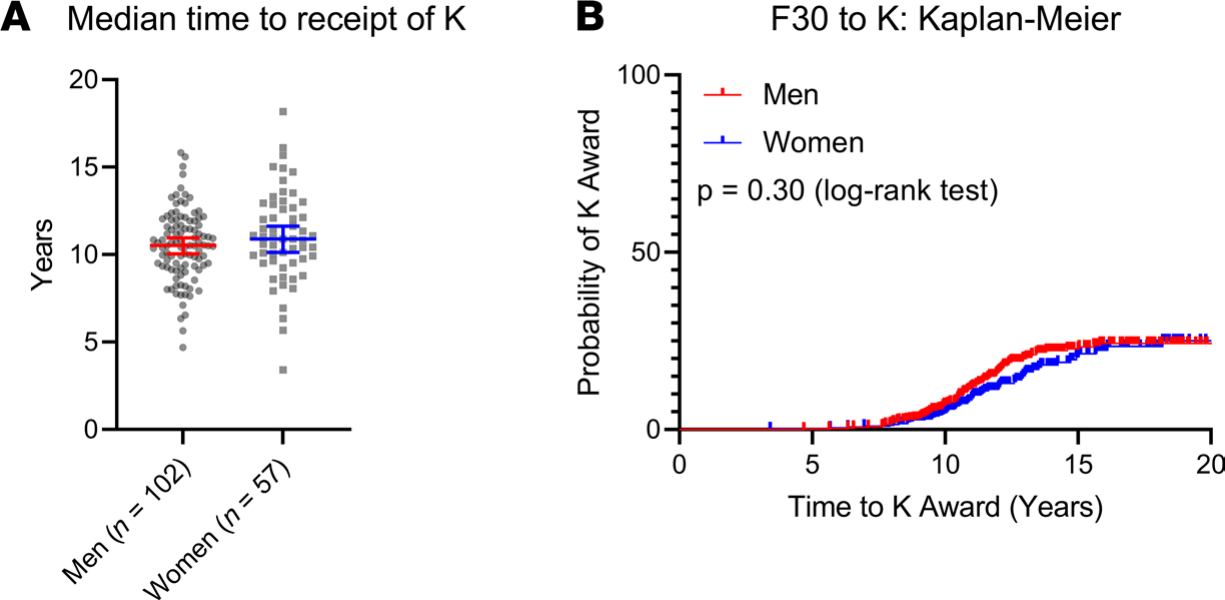
Transition from F30-to-K award. (**A**) Of *n* = 1015 F30 awardees between 1990 and 2012, *n* = 102 men and *n* = 57 women went on to receive a K award in the 11.9-year median follow-up period. Overall median time to the start of the K award from the start of the F30 award was 10.7 years (10.5 years for men, 10.9 years for women). Bars represent median with 95% CI. (**B**) In the time-to-event analysis, no difference existed between men and women in the probability to receive a K award (*P* = 0.30, log-rank testing).

For those who received a K award, the overall median time from the start of the F30 to the start of the K award was 10.7 years (men, 10.5 years; women, 10.9 years) (Figure 3A). In the Kaplan-Meier time-to-event analysis, there was no difference between men and women in probability of receiving a K award (*P* = 0.30, log-rank testing) (Figure 3B).

### F30-to-R analysis.

F30 grantees awarded between 1990 and 2007 were included in the F30-to-R analysis. Over this time period, there were 433 F30 awardees, 162 (37.4%) of whom were women and 271 (62.6%) of whom were men. For this cohort, with a subsequent median follow-up period of 16.6 years from the start of F30 funding, we found that 101 (23.3%) proceeded to receive a K award, with a median time to start of the K award at 11.0 years. The success of women (21.0%; 34 of 162) and men (24.7%; 67 of 271) receiving K awards within this R cohort was comparable (*P* = 0.41, Fisher’s exact testing). Of these 101 K awardees, 48 went on to receive an R award, indicating a K-to-R conversion of 47.5% (women, 29%; men, 56%; K-to-R conversion).

Independent of the K award mechanism, an additional 32 individuals who had F30 awards went on to receive R awards, bringing the total F30-to-R conversion rate to 18.5% (80 of 433). The F30-to-R award recipients included 15 women (18.8%) and 65 men (71.2%). With normalization, 9.3% of women who received an F30 award went on to receive an R award (15 of 162); 24.0% of men who received an F30 award went on to receive an R award (65 of 271) (*P* = 0.0001, Fisher’s exact testing).

**Figure 4:**
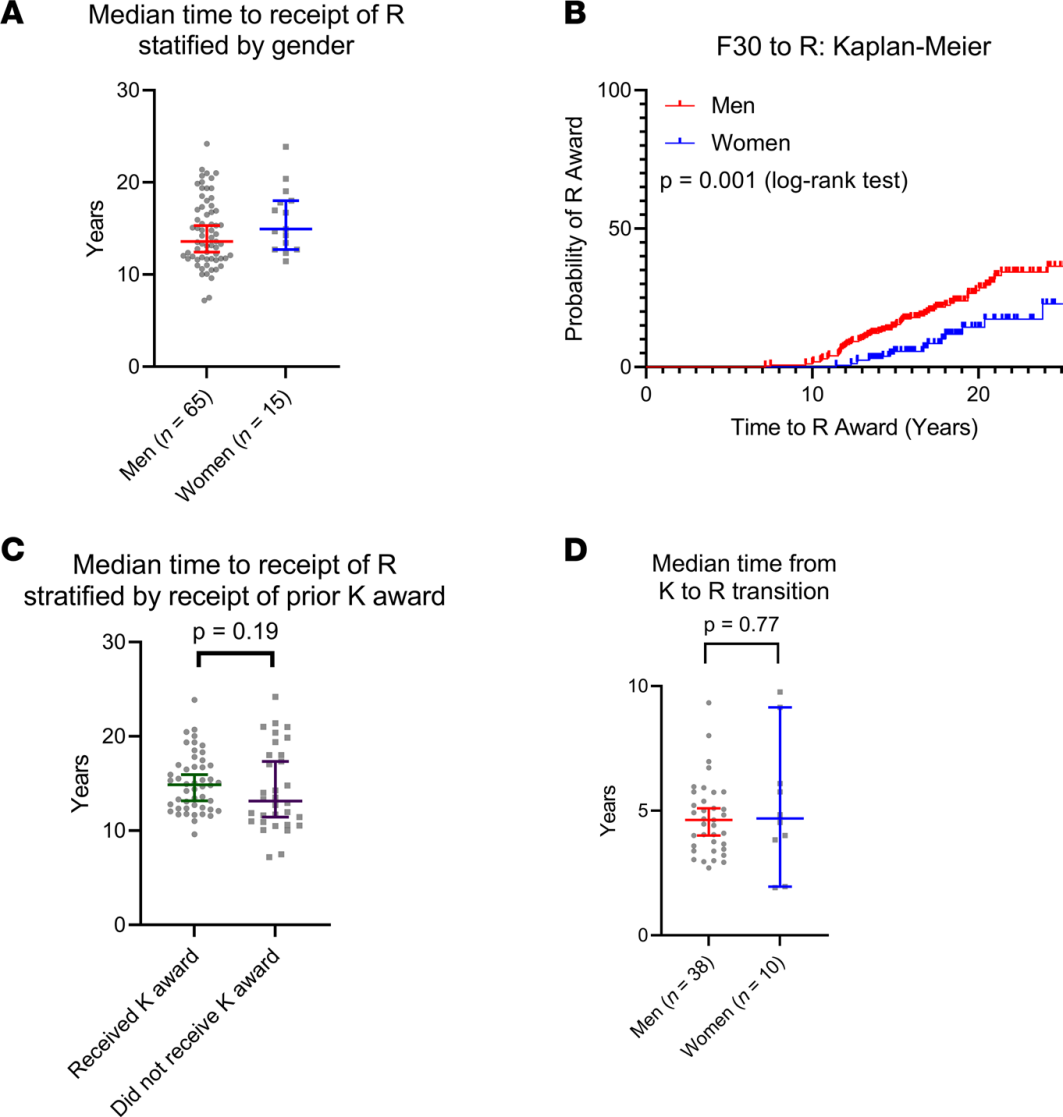
F30-to-R award. (**A**) Of *n* = 433 F30 awardees between 1990 and 2007, *n* = 65 men and *n* = 15 women went on to receive an R award in the 16.6-year median follow-up period. Overall median time to the start of the R award from the start of the F30 award was 14.2 years (13.6 years for men, 14.9 years for women). (**B**) In the time-to-event analysis, men were significantly more likely than women to secure R funding (*P* = 0.001, log-rank testing). (**C**) Of the 80 R awardees, 48 had received a prior K award; there was no difference in the median time to the receipt of R stratified on whether or not the recipient had a prior K award (*P* = 0.19). (**D**) Of the 48 R awardees that had a prior K (*n* = 38 men, *n* = 10 women), there was no difference in median time from K to R transition (defined as the start of K to the start of R) (*P* = 0.77). Displayed bars represent median with 95% CI.

For those who received an R award, the overall median time from the start of the F30 to the start of the R award was 14.2 years (men, 13.6 years; women, 14.9 years) (Figure 4A). In the Kaplan-Meier time-to-event analysis, women were significantly less likely to receive R awards over the analysis period (*P* = 0.001, log-rank testing) (Figure 4B).

There were no differences when looking at the median time to R award from the start of the F30 when comparing those who did and did not receive a prior K award (median time to R for those who received a K award, 14.9 years; median time to R for those who did not receive a K award, 13.1 years; *P* = 0.19) (Figure 4C). For R recipients who did receive a prior K award, there were no differences between men and women in terms of median time from start of the K to start of the R award (median for men, 4.6 years; median for women, 4.7 years; *P* = 0.77) (Figure 4D).

### Results in the context of other studies.

While F30 recipients’ overall success rate in obtaining K awards was lower than we expected, we found a 48% increase in the proportion of K grant success (23.3% vs. 15.7%) in the cohort followed for a median of 14.2 years versus those followed for 10.7 years, suggesting that the length of the training period for physician-scientists delays achievement of this milestone. The rates of K awards for MD-PhD F30 recipients are comparable to or exceed those reported elsewhere for MD-PhDs (4, 7) (Table 1). Rates of R funding for F30 awardees were comparable to those reported by Harding et al. for MSTP graduates from 1990 to 1999 based on NIGMS data but were less than those in an earlier (1980–1989) cohort (4). Self-reporting of R grants by MSTP graduates with full-time academic/research positions (7) was also higher than in our F30 awardee cohort (Table 1). In the report by Akabas and Brass (7), which had a 64% survey response rate, 3081 of 6981 respondents held current positions in academia or in research institutes, and K and research project grant (RPG) success was reported for this group. It is notable that, in survey-based studies, responders are significantly more likely to have received R01 funding compared with nonresponders (8). If the responding MD-PhDs in Akabas and Brass who did not hold a current academic or research position had lacked prior RPG research funding, then the overall RPG success of respondents in that report (7) would have equaled that in our study (see calculation included in Table 1; this would be a lower limit for K and RPG attainment by the full self-reporting cohort).

We could not identify literature analyzing future NIH funding for PhD-only predoctoral NIH grantees to compare with that of the MD-PhD cohort. In order to estimate this, we analyzed K and R funding for individuals receiving F31 awards over the same periods of time that we analyzed for MD-PhD trainees receiving F30 funding. F31 grants are predoctoral NRSA grants that fund graduate work only, unlike F30 awards, which are restricted to dual-degree candidates and fund both graduate and medical school stipends. As such, the large majority of MD-PhD trainees use the F30 rather than F31 mechanism, save for those applying to institutes (National Institute of Neurological Disorders and Stroke [NINDS] and National Institute of Arthritis and Musculoskeletal and Skin Diseases [NIAMS]” to define “NINDS” and “NIAMS) that do not participate in the F30 award mechanism. While some MD-PhDs are therefore included in the F31 pool, the great majority of F31 awardees are PhD-only trainees. Given the larger number of PhD trainees, 14 times more F31 grants than F30 grants were awarded in 1990–2007, and roughly 12 times more F31 than F30 grants were awarded in 1990–2007. Given the challenge of manually curating this larger grant portfolio, we report only total numbers and not a gender breakdown for future grants for the F31 cohort (see Methods).

Compared with future grants received by F30 recipients, we observed a much smaller percentage of F31 recipients receiving K awards but a larger proportion receiving R awards (Table 1).

These data may overestimate the number of K and R awards obtained by the PhD-only cohort, given the inability to remove MD-PhD F31 recipients from the pool (the degree program of NINDS F31 applicants is not tracked; Stephen Korn, NINDS, personal communication).

## Discussion

A central feature in the training of MD-PhD candidates is the development of skills in planning and writing compelling predoctoral grants to the NIH to be funded under the Ruth Kirschstein NRSA F30 mechanism. Garnering F30 support reflects a mastery of scientific thinking and communication, because successful proposals require demonstration of strong foundational knowledge in the area of the proposed project and framing of rigorous investigative plans. The current study captured all MD-PhD F30 awardees from 1990 to 2012 and tracked individual-level funding trajectories to uncover trainee-specific and time-dependent award histories. We expected that the subset of MD-PhDs who had demonstrated ability to earn extramural funding early in their training could be expected to parlay this experience at later points in their career.

We utilized the F30 funding mechanism to identify dual-degree MD-PhD trainees for this study. From this cohort, a minority of F30 awardees went on to obtain K award (15.7%) or R award (18.5%) funding, with median follow-up of 10.7 and 14.2 years, respectively. This indicates that while F30 funding may benefit future success in procuring grants, it is insufficient for garnering K- and R-level grant support from NIH. Whether F30 funding increased the success rate of future R01 submissions cannot be judged, because data on total applications by MD-PhD trainees were not accessible to us. We can conclude, even for this elite cohort of predoctoral physician-scientist F30 awardees, that gender disparity manifests at the level of independent funding. Men and women obtained (mentored) K grant funding at equal rates; however, the success of women in obtaining independent R grant funding was only 40% of that of their counterparts who were men. For those F30 awardees who did get a K award, the subsequent conversion rate to an R award was 47.5%; the conversion rate showed a substantial gap between the genders (29% K-to-R conversion rate for women compared with a 56% conversion rate for men).

These findings bring about questions of whether F30 grants help to train the next generation of physician-scientists. Two issues are worth noting. First, R01 success may be an inadequate measure of the value of successful predoctoral grant preparation and submission. A large proportion of MD-PhD program graduates support their research with foundation and/or pharmaceutical company money rather than NIH grants, as noted in Brass and Akabas (7).

Notably, physician-scientist training allows trainees to garner skills that are attractive for several career paths and clinical/translational contributions independent of the RPG measure. The scope of activities in which physicians contribute to discovery is broad and increasing.

Second, the years captured in our study could underestimate the linkage between F30 receipt and future funding. The majority of F30 grants were awarded after the window considered in this study, and the overall effect of F30 receipt on independent grant acquisition is not yet evident for most recipients. Our cohort also preceded the broad implementation of K99/00 and similar transitional funding mechanisms. Even for those captured in this study, the follow-up period may be inadequate given the extended training required of physician-scientists currently and the effect of clinical commitments on the pace of productivity. Curriculum vitae analysis would help determine whether F30 recipients move from academic medicine into other careers.

Almost half of those who did receive an R award, in this F30 cohort, did not receive an antecedent K award. Multiple factors could contribute to this finding, including a decision by MD-PhDs to forego this opportunity in order to limit training duration, institutional pressure to forego the smaller grant (e.g., in procedural specialties), or less emphasis on the K award in the earlier MD-PhD cohort that we targeted for R award analysis. For a subset of F30 awardees, pursuit of a K award may be perceived as too costly an extension of the training period. The need to shorten training time was noted as one of the main action items by the 2014 Physician-Scientist Workforce Working Group. This group suggested shortening the time to independent research by 5 years (14, 15).

K awards were rarer among the primarily PhD-only recipients of predoctoral F31 training grants. This likely reflects the limited use of K awards by the NIH for PhD scientists (whereas the K08 and K23 mechanisms are limited to clinicians), as well as the fact that the major K award used, the K99, was introduced late in the capture period. Notably, the percentage of R awards was higher in the F31 recipient pool (similar to the rate among male F30 recipients in the 1990–2007 cohort). This could be a manifestation of the higher reliance on independent R awards early in the academic PhD career and/or a shorter training period compared with MD-PhDs. While beyond the scope of this study, it is conceivable that receipt of F31 grants by PhD trainees tracks with persistence in academic careers.

Our finding of a funding discrepancy between male and female MD-PhDs occurring during the K-to-R transition is not without precedent. Previous work into the funding rates of physician-researchers with K awards showed significantly lower R01 funding rates for women compared with men (8, 9), and this gap is evident in a recent analysis of funding success of MD and/or PhD T32 recipients (16). A possible explanation for the gender gap in R-level funding of MD-PhDs is implicit bias at the level of NIH funding. However, gender-biased handling of MD-PhD applications may not account for this gap. In 2014, the NIH Physician-Scientist Workforce Working Group, in coordination with the American Association of Medical Colleges (AAMC), published a comprehensive physician-scientist workforce report. Notably, they reported no difference in the R grant award rate for men (24.6%) and women (24.8%) MD-PhDs in 2012, or gender differences in persistence in resubmitting initially rejected R grants (17). Although studies using data-driven text mining of R01 renewal applications identified wording compatible with gender bias in concert with lower criterion scores (18, 19), a study of experimental initial R01 review using modified names throughout the applications did not uncover bias (20). Moreover, the self-reported success rate for academic male and female MD-PhDs was comparable (78% vs. 73%) (7). The low K-to-R rate for female MD-PhD F30 awardees could indicate a disproportional decrease in applications by female MD-PhDs with or without continuation in the physician-investigator pipeline. Although NIH application rates for individual MD-PhDs were unavailable to us, a 2011 study of extramural funding using application-based and person-based metrics found that, for K08 and K23 awardees, the rate of applications for subsequent R01 support was greater among men than women (21).

Discrimination against women in science fields has been well documented and is relevant to female grantees in our MD-PhD cohort. Reports in the 1990s and early 2000s described the overt discrimination against women with equal qualifications to colleagues who were men. To address the reason for the disparity in women attaining funding and senior faculty positions at academic institutions that we continue to see today, Ceci and Williams analyzed discrimination against women in journal reviews, grant funding, and hiring in math-intensive and science fields (22). The authors found that women fare as well as men in these areas if given equal access to resources. However, women often occupy positions that do not have equal access to resources due to factors concerning gendered expectations and concerns surrounding family formation, childrearing, lifestyle choices, etc., that often lead to more leaves of absence from graduate medical education (22, 23). A more specific study of women in the field of obstetrics and gynecology, a field many women have entered since the 1990s, shows that gender wage gaps, academic advancement, and attainment of leadership roles remain a challenge (24). These studies reveal that there are systemic factors that contribute to a disparity in women attaining funding or advancement in their fields through unequal access to resources. As described in 2008 by Ley and Hamilton, although there has been a “pull” to attract and recruit women into science fields as trainees, there has not been an effective “push” that reduces attrition along the career development track (25).

The process of generating a successful predoctoral NIH grant acquaints MD-PhD trainees with the mechanics and critical thinking involved in future grant submissions but is insufficient preparation on its own for future independent NIH funding. Moreover, the inability to procure R-level funding contributes to attrition in the physician-scientist pipeline. Persistence requires both extrinsic progress (e.g., grants submitted and awarded, publications, presentations, academic advancement) and intrinsic career factors (e.g., satisfaction, efficacy, resilience) (26, 27).

Contributory barriers to persistence and funding success as a physician-scientist include difficulty in balancing clinical and research responsibilities and professional and personal life needs (28–31). At our institution, the University of Pittsburgh Medical Center, residents and fellows in the academic track viewed the balancing of clinical and research responsibilities and the challenge of procuring funding as equal impediments to physician-scientist careers (61% vs. 58%, respectively; our unpublished observations).

A better understanding of attrition in grant acquisition by those receiving predoctoral NIH support can illuminate whether the leakiness in the physician-scientist pipeline reflects informed syntonic choices or disruption of careers by funding gaps. Steps to sustain physician researchers extend beyond scientific and professional development skills. Among faculty, time-banking interventions to offset work-work and work-life time conflicts have been reported to yield sizable benefits (32). Career coaching can bolster the ability of physicians to align their time commitments with their priorities, reframe obstacles as opportunities, forge a practical career management approach with accountability, and highlight their value to the institutional mission (33, 34). Importantly, such coaching initiatives lower attrition (35, 36). Funding bodies and academic institutions have started to implement strategies to address the disparities in gender equity. In a critical first step, the NIH has recently released an announcement that it will cover a portion of childcare costs for NRSA individual fellows. This initiative joins institutional strategies to achieve and support equity between men and women in academic medicine (37, 38). Finally, follow-up studies are needed that look at the intersection of sex and race in achieving funding along the physician-scientist training pipeline so that strategies that address this intersectionality are implemented.

### Limitations of this study.

Our individual-level data were obtained from public funding records, and information at that level on application rates for K and R awards was not available to us. This limits our ability to convincingly parse contributory factors to the observed overall and gender-specific award rates. An additional limitation was that the distribution of F30 awards was left skewed in our data set. While the first F30 was awarded in 1990, the majority of F30 awards have been awarded beyond 2010. Therefore, our analysis here captures the first generation of dual-degree physician-scientists who received funding through this mechanism but does not capture the majority of those who have received this award. Subsequent analyses must be performed to verify whether the trends seen here persist through the current generation of early career physician-scientists who are applying for K or R awards. Additionally, a subset of early F30 awardees (1990–2007 cohort) will continue to obtain their first R grants after the cutoff set for analysis in this paper.

With ongoing first R funding for this group, it is premature at this point to conduct definitive qualitative analysis of characteristics (including any role for gender, interim foundation funding, specialty) associated with delayed first R grant acquisition.

Since their conception in the 1950s, there has been substantial growth in the numbers of MD-PhD programs. As of 2016, there are 90 MD-PhD programs, 45 of which are funded by the NIH through MSTP (T32) awards (4). The creation of MSTP programs underscores the recognized need to train physician-scientists. While these programs have evolved increasingly comprehensive training platforms, MD-PhD programs cannot substitute for ongoing guidance and training throughout the postgraduate and early faculty stage. We need strategies to build a more robust continuum of research and resilience extending from the predoctoral through the independent investigator stage. MD-PhDs have shown that they can frame compelling grant applications early in their career. In order to leverage this skill successfully in the interest of discovery, an equally robust curriculum should be grounded in the elements needed to navigate the academic landscape successfully and equally for women and men.

## Methods

### Data sources and acquisition.

F30 recipients were identified through the NIH’s Research Portfolio Online Reporting Tools (RePORT). F30 data were downloaded for each year from 1990 through the end of 2019 (data was accessed in December 2020). Each year’s F30 grantees were collated into a single data file. We focused specifically on MD-PhD dual-degree predoctoral grantees and removed DVM/PhD, DDS/PhD, and AuD/PhD F30 recipients from the analysis. Additionally, because F30 grants span multiple years, grantees are listed in RePORTER for each year of funding. We therefore removed duplicate entries and only kept the individual grantee entry that corresponded with the start of their F30 grant.

To determine if and when F30 recipients advanced to receive K- or R-level NIH awards, we manually searched each F30 recipient on NIH RePORTER to determine (a) if they received a K- or R-level award, (b) the specific type of K or R award and their equivalents, and (c) the time to award, calculated as the start of the F30 award to the start of the K and/or R award. K awards and equivalents included the following award mechanisms: K01, K02, K08, K22, K23, K38, and K99. R awards and equivalents included the following award mechanisms: R01, R03, R21, R34, R35, R43, and R56, with the R01 mechanism constituting the majority (70%) of awards. When we allude to K or R awards, we are including all of these mechanisms. The follow-up period lasted from the start of the F30 grant to either the start of the K or R award or until the date of final data collection (May 1, 2021).

As gender was an important variable in this analysis, two authors independently conducted internet searches to attribute gender for the F30 recipients on the basis of text from institutional websites, use of gender-specific pronouns, or an explicit statement of gender, which is consistent with methods used by prior publications (8, 9). In no cases were there discrepancies between reviewing authors. To account for surname changes among women, we investigated all F30 recipients who were women who did not receive a subsequent K or R award. After searching institutional websites for potential names changes, we do not believe the lack of subsequent grant funding to be a result of name changes for any of the F30 recipients. Prior publications have found very low rates of name changes in this setting (9).

We also analyzed the success of F31 grant awardees (primarily PhD-only candidate programs) in obtaining future NIH K or R funding. The pool of F31 awardees is over 10 times greater than that of F30 recipients, making manual curation impractical. We used the RePORTER database functionality rather than manually curating the grant trajectory of F31 awardees. Those receiving F31 grants between 1990 and 2007 and between 1990 and 2012 were identified through searching RePORTER, and their PI IDs were collected and used to search all associated awards for each cohort up to 2021. Awards were filtered for unique listings (the initial year) to remove multiple award listings from successive years. Manual curation of a subset (1100) of results showed that 1.9% of PIs were assigned multiple PI IDs (generally middle name/initial). Additionally, awarded grant results contained co-PIs who had not received a prior F grant (8.9% of unique PI listings). We considered double ID listings and co-PI listings to constitute false positives and reduced the level of K awards ascribed to the entire F31 cohort by 1.9% and that of R awards (in which co-PI’s appear) by 10.8% (1.9% + 8.9%). Applying this search strategy to our F30 cohort gave results consistent with the manual F30 search.

### Main outcomes.

To account for differences in time to grant receipt, we analyzed K and R awards in the following manner. For the F30-to-K award analysis, we analyzed all individuals who received an F30 award between 1990 and 2012. For F30-to-R award analysis, we analyzed all individuals who received an F30 award between 1990 and 2007 (summarized in Figure 1). The earlier F30 endpoint for R analysis reflects the longer time span needed to achieve R funding. Within the F30-to-R award analysis, we also determined which grantees received a K award between the F30 and R award, allowing for F30-K-R and for F30-R longitudinal analysis. Primary outcomes included proportions of F30 recipients receiving K and R awards stratified by gender as well as the time to award for men and women.

### Statistics.

We analyzed the data using Stata v.15. We used Fisher’s exact test to determine if proportional differences existed between men and women who received K and R awards. We used the Kaplan-Meier method to estimate the probability of the receipt of K and R awards and the log-rank test to assess differences between men and women. Receiving a K or R award through May 2021 was considered the event of interest for F30 recipients. Displayed bars in figures represent the median with 95% CI. *P* values of less than 0.05 were considered significant.

### Study approval.

This retrospective cohort study was exempt from institutional review board approval, as all data are publicly available.

## Author contributions

CRK, NC, SGC, and RAS designed the study. NC acquired the data. NC, RAS, SGC, and SMN performed data analysis and determined statistical plan. CRK, NC, RAS, and SGC drafted the manuscript. CRK, NC, RAS, SGC, and SMN provided critical revision of the manuscript.

## Figures and Tables

**Table 1:**
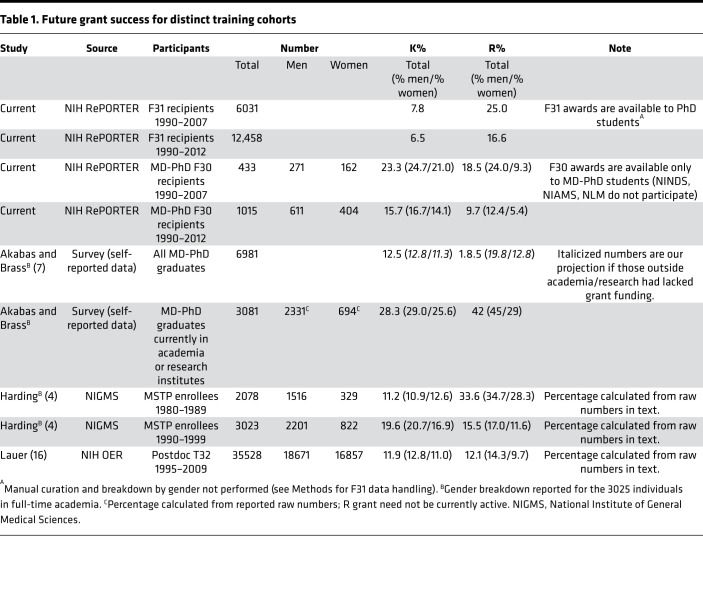
Future grant success for distinct training cohorts

## Version 1 03/22/2022

Electronic publication
